# Statistical Significance of Precisely Repeated Intracellular Synaptic Patterns

**DOI:** 10.1371/journal.pone.0003983

**Published:** 2008-12-19

**Authors:** Yuji Ikegaya, Wataru Matsumoto, Huei-Yu Chiou, Rafael Yuste, Gloster Aaron

**Affiliations:** 1 Laboratory of Chemical Pharmacology, Graduate School of Pharmaceutical Sciences, The University of Tokyo, Tokyo, Japan; 2 Precursory Research for Embryonic Science and Technology, Japan Science and Technology Agency, Tokyo, Japan; 3 Department of Biological Sciences, Howard Hughes Medical Institute, Columbia University, New York, New York, United States of America; 4 Biology Department, Neuroscience & Behavior Program, Hall-Atwater & Shanklin Labs, Wesleyan University, Middletown, Connecticut, United States of America; Max-Planck-Institut fuer Neurobiologie, Germany

## Abstract

Can neuronal networks produce patterns of activity with millisecond accuracy? It may seem unlikely, considering the probabilistic nature of synaptic transmission. However, some theories of brain function predict that such precision is feasible and can emerge from the non-linearity of the action potential generation in circuits of connected neurons. Several studies have presented evidence for and against this hypothesis. Our earlier work supported the precision hypothesis, based on results demonstrating that precise patterns of synaptic inputs could be found in intracellular recordings from neurons in brain slices and *in vivo*. To test this hypothesis, we devised a method for finding precise repeats of activity and compared repeats found in the data to those found in surrogate datasets made by shuffling the original data. Because more repeats were found in the original data than in the surrogate data sets, we argued that repeats were not due to chance occurrence. Mokeichev et al. (2007) challenged these conclusions, arguing that the generation of surrogate data was insufficiently rigorous. We have now reanalyzed our previous data with the methods introduced from Mokeichev et al. (2007). Our reanalysis reveals that repeats are statistically significant, thus supporting our earlier conclusions, while also supporting many conclusions that Mokeichev et al. (2007) drew from their recent *in vivo* recordings. Moreover, we also show that the conditions under which the membrane potential is recorded contributes significantly to the ability to detect repeats and may explain conflicting results. In conclusion, our reevaluation resolves the methodological contradictions between Ikegaya et al. (2004) and Mokeichev et al. (2007), but demonstrates the validity of our previous conclusion that spontaneous network activity is non-randomly organized.

## Introduction

In mammals, the sensory neocortex is often considered as the highest level of sensory processing, both in an anatomical and functional hierarchical sense. Many studies have examined the neocortical response to sensory input in individual neocortical neurons, and how that response is transformed in different cortical layers. In contrast, the present study examines *spontaneous* activity in single neurons in primary sensory neocortex, that is, the activity of a single neuron when no extrinsic stimulation is given. Perhaps surprisingly, many neurons in primary sensory cortex fire action potentials even during the absence of any sensory stimulation [1]. This phenomenon may be less surprising in light of the fact that most synaptic connections in neocortex originate from other neocortical neurons, and most neocortical neurons receive no direct synaptic input from the thalamus [2]. In this sense, neocortical activity is largely generated intrinsically, albeit with an important modulation from thalamus [3], [4]. Indeed, studies have demonstrated that patterns of neocortical activity during sensory stimulation are very similar to patterns seen without sensory stimulation [5]. Interestingly, the similar result can also be seen in a slice preparation that preserves thalamocortical connections between ventrobasal thalamus and somatosensory cortex; in this study, patterns of spontaneous cortical activity can be found that are significantly similar to patterns generated by thalamic stimulation [6].

Such studies suggest that the neocortex is a pattern generator, producing patterns of activity regardless of whether patterned stimulation is presented. One way to investigate this hypothesis is to examine relatively long stretches of neocortical spontaneous activity, looking for repeating motifs of activity, in either spike trains or intracellular recordings. With the help of computationally-intensive searches, there have been several studies that have claimed to demonstrate the existence of surprisingly precise and intricate patterns of repeating activity in neuronal circuits from *in vivo* preparations [7]–[13], intact slices [1], [7], [14], [15], dissociated neuronal cultures [16]–[18], and sophisticated neuronal models [19]–[21]. In addition, these results have been supported by persuasive studies that argue for the existence of such repeating patterns [22].

However, the interpretation of these findings has been contentious, as there is no universally accepted method for demonstrating whether a precisely repeating pattern is randomly generated versus deterministically produced. In addition, there have been persuasive studies arguing that such patterns could be randomly generated [23]–[25].

**Figure 1:**
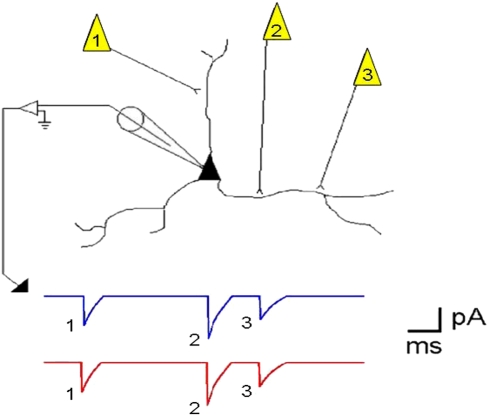
A cartoon illustrating how repeats of action potential sequences in a cortical network can be recorded in a single neuron. The picture depicts a pyramidal neuron being recorded with an intracellular electrode that measures postsynaptic currents (PSCs) during voltage clamp recordings. A series of action potentials in three neurons forming synapses with the neuron can be recorded. The blue trace represents such a sequence that was recorded, and the red trace shows the same sequence repeating at some later time in the recording.

One obstacle to the study of these network patterns is the limited ability to record the participating neurons. That is, many neurons should be simultaneously recorded with a high temporal precision in order to increase the probability of detecting a network phenomenon. To overcome this problem Ikegaya et al. (2004) [7] introduced a template-matching search program for single intracellular recordings. The rationale of the technique was that since a single neuron receives 100 s to 1000 s of synaptic inputs from different neurons, then it is conceivable that a single neuron could serve as a “microphone” of the neuronal network (Fig. 1). In this study, we found remarkable examples where the intracellular currents recorded in a single neuron were seen to repeat with millisecond precision. Such examples do not mean that these phenomena could not emerge by chance alone. To examine the null hypothesis of stochastic generation of precise repeats, surrogate data were generated and compared to the original data. More putative repeats were found in the original data than in the surrogate data, and so the null hypothesis of stochastic generation was rejected for these recordings.

Mokeichev et al. (2007) [21] proposed three new methods for creating surrogate data of intracellular traces, and their results did not reject the null hypothesis that repeats of cortical activity are stochastically generated. This conclusion was supported by the finding that surrogate data sets, based on the original data but randomly shuffled, contained as many repeats of activity as the original data. Their detector program for finding repeats was the same as that created in Ikegaya et al. (2004) (although translated to a different language, Matlab to C++). The notable contribution of the paper was the introduction of three additional surrogate data generation techniques, each designed to test the null hypothesis for stochastic generation of repeats. Most of their analyses were conducted on five intracellular traces obtained from rat cortex, *in vivo*. We also conducted *in vivo* mouse cortex recordings, similarly to Mokeichev et al. (2007), and reproduced their results: in these recordings we could not reject the null hypothesis of stochastic generation for repeats (data not shown).

However, the original data analyzed in Ikegaya et al. (2004) yielded contrary results when analyzed with the newer Mokeichev et al. (2007) algorithms. The data examined in Ikegaya et al. (2004) and Mokeichev et al. (2007) overlap only with regards to cat *in vivo* recordings, originally recorded in Lampl et al. (1999) [26]. There are noticeable qualitative differences between the cat *in vivo* data, mouse *in vitro* data, and the rat *in vivo* data, and we believe these differences can account, perhaps partly, for the different results. Here we show that two of the shuffle tests (phase randomization and Poisson firing model) produce surrogates that contain many fewer repeats than the original traces in the mouse *in vitro* and cat *in vivo* data. In addition, we show that the results from the third shuffle test (interval shuffling) require closer consideration of the detector program itself in order for correct conclusions to be drawn. We also demonstrate that the detector program used in both Ikegaya et al. (2004) and Mokeichev et al. (2007) is insufficiently sensitive to determine that an artificial data set, with many precise repeats implanted by the investigator, can be distinguished from surrogate data. We explain the defects in the original detector program, and then address those defects with the creation of an improved repeat detector program that can distinguish the implanted data from its surrogates. We use the improved detector program to demonstrate that the original data from cat *in vivo* recordings contain more repeats than those from the surrogate shuffle data. Finally, we demonstrate that recording conditions have a significant effect on repeat detection, and this effect may explain the set of contrary results obtained in this study.

After these careful re-analyses, we conclude that the temporal profile of a series of synaptic inputs into a neuron from the surrounding network is organized to a degree that cannot be explained by chance.

## Results

### Searching for repeated patterns of synaptic inputs

Before revealing our results, we remind the reader of the goal of these experiments: we are exploring whether patterns of post synaptic potentials/currents (PSP/Cs) recorded in a single neuron recur with a frequency and precision beyond that would be expected by chance occurrences. This exploration occurs through a series of tests where we take our original data and shuffle it in various ways, producing surrogate data that is identical to the original except for a rearrangement in the order of events or frequencies. These surrogates are then compared against the original. We argue that the interval shuffling technique from Mokeichev et al. (2007) is a significant advance and produces surrogate data that are much more closely preserved versions of the original data—so much so that our original detector would not be equipped to detect the difference, even if the difference was significantly real. We demonstrate this lack of sensitivity with artificially implanted repeats, and then produce a new detector that is better equipped for the task of detecting these repeats. This new detector can then satisfy all the surrogate tests produced thus far, and supports the rejection of the null hypothesis of stochastic generation of repeats.

**Figure 2:**
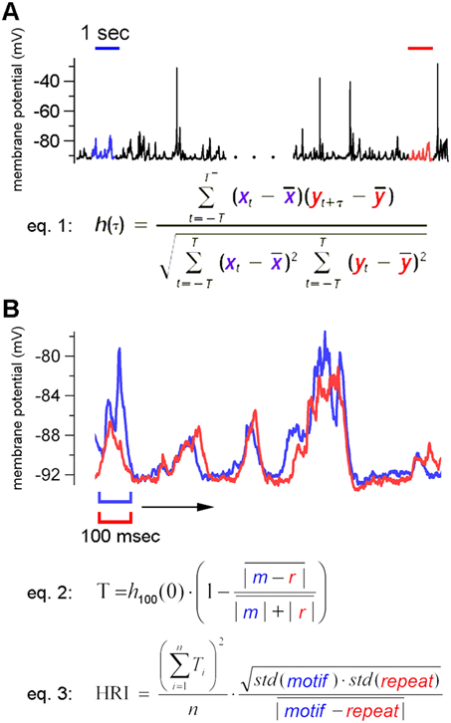
Repeat detection via LRI-HRI search. This briefly describes the method for repeat detection used in both Ikegaya et al. (2004) and Mokeichev et al. (2007). (A) LRI search. The entire recording is searched with a nested loop template matching algorithm where each one second interval is compared with nearly every other one second interval using cross-covariance. If the cross-covariance measured between two 1 second segments is beyond a set threshold, then the respective intervals are saved for a subsequent analysis. In this figure, two such segments are highlighted, indicating the motif (blue) and subsequent putative repeat (red). (B) The captured segments from the LRI search above are aligned, superimposed and analyzed with an HRI scan. A 100 msec window, the estimated length of an average PSP, is used to compare all 100 msec intervals between this motif-repeat pair, again using cross-covariance (*h* function), normalized by the respective amplitudes of the intervals (eq. 2). The final HRI is then computed (eq. 3).

Mokeichev et al. (2007) and Ikegaya et al. (2004) used essentially the same detector program, called here the LRI-HRI program, to find repeating patterns of membrane potential fluctuations in cortical intracellular recordings (Fig. 2). The same program is used here in the 1^st^ half of the manuscript. The key feature of the program is the two stage construction: the first stage, Low Resolution Index (LRI), compares all 1 second intervals against all other 1 second intervals using a nested loop, template matching algorithm with cross-covariance as the basis for comparisons (Fig. 2). This is a rough way of finding segments of the recording that may be similar to each other, and the location of these segments are saved for the subsequent High Resolution Index (HRI). HRI examines the 1 second intervals indicated by LRI using 100 msec comparison windows. The 100 msec is roughly matched to the length of the average PSP in the recording. In contrast, the 1 second window used in the LRI was chosen arbitrarily and isn't necessarily matched well for putative motif-repeats, a problem discussed later in the manuscript. Despite such problems, the LRI-HRI program can find many convincing motif-repeat pairs (Fig. 2B, see also Ikegaya et al. (2004) and Mokeichev et al. (2007) for many examples).

Similarly to Mokeichev et al. (2007), we conducted *in vivo* patch-clamp recordings from layer 2/3 pyramidal cells in somatosensory or motor cortex of anesthetized mice. The cells were intracellularly labeled with biocytin and morphologically identified *post hoc*. We produced three different types of surrogate traces in the same manner as in Mokeichev et al. (2007): (1) phase randomization surrogates, created by shifting oscillatory phases in the Fourier transform; (2) Poisson implanted PSCs, created by implanting artificial postsynaptic currents (PSCs) in “plain” mother traces; (3) 400 msec shuffled surrogates, created by cutting the original waveform into 400 msec intervals, and then randomizing the order of those intervals. Using the LRI-HRI program, no significant motif-repeats were found in these recordings; that is, we failed to find a significant difference in the number of motif-repeats between original and randomized traces (data not shown), replicating the results of Mokeichev et al., (2007).

**Figure 3:**
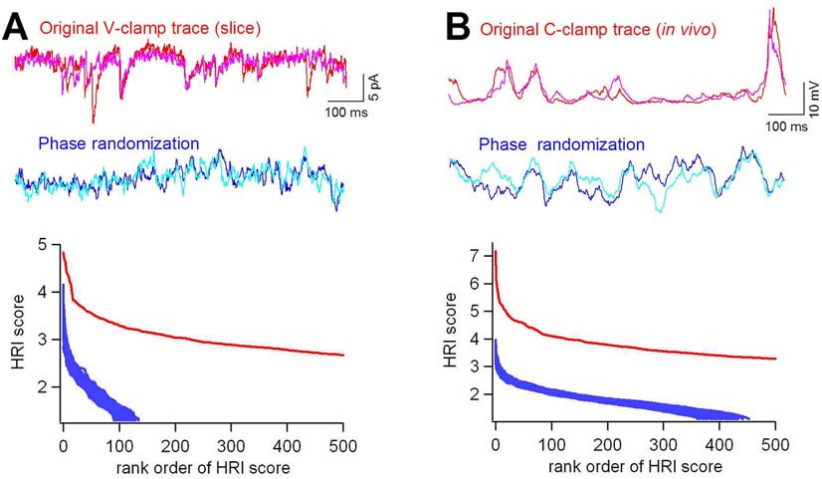
Phase randomized surrogates display many fewer repeats than original traces. (A) An 8-min voltage-clamp trace recorded from a layer 5 pyramidal cell in a mouse visual cortex slice (red) was compared to fifty surrogates generated by phase randomization (blue). Trace segments that gave the highest HRI values are shown in the top panels, and the HRI sores of all detected segments are shown in the bottom panel after rank sorting. In the original trace, more segments passed the LRI threshold, and their HRI scores are higher, as compared with phase-shuffled surrogates. (B) The same analysis was conducted on a 3-min current-clamp recording *in vivo* from an anesthetized cat, producing similar results.

We then analyzed the traces used in Ikegaya et al. (2004), using the same methods, and report nearly opposite results with regards to phase randomization surrogate trace generation and Poisson PSC surrogates. These recordings consisted mainly of intracellular voltage-clamp recordings from layer 5 pyramidal cells of mouse primary visual cortex slices, *in vitro*. The repeats found in the original recording showed higher HRI values than any of the 50 surrogates created via phase randomization (Fig. 3A). We next re-tested the same *in vivo* current-clamp recording from a neuron in primary visual cortex of anesthetized cats, the same data used in Ikegaya et al. (2004). Using phase randomization surrogates, there were clearly more motifs found in the original trace, rejecting the null hypothesis for stochasticity (Fig. 3B). This does not conflict with findings from Mokeichev et al. (2007) as this test on cat *in vivo* data was not reported in that study.

**Figure 4:**
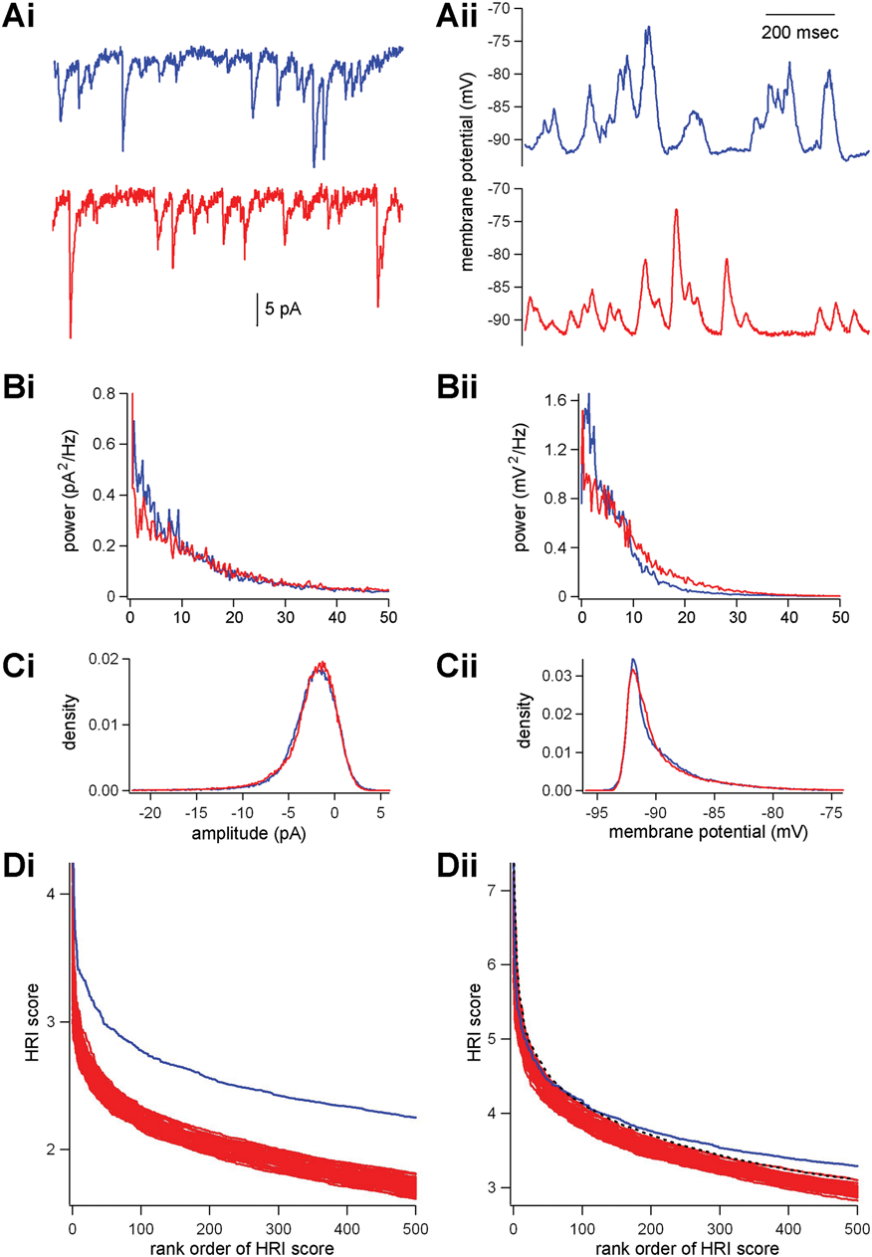
Poisson-generated surrogates display significantly fewer repeats than original traces. (Ai) An 8-min voltage-clamp trace recorded from a layer 5 pyramidal cell from mouse visual cortex (blue, top) was mimicked by a Poisson process that produced a surrogate trace (red, bottom). (Aii) The same procedure was conducted on a 3-min current-clamp recording *in vivo* from an anesthetized cat, producing a Poisson-generated trace (red, bottom) modeled from the original (top). (B and C) Using an error-minimization algorithm, the variables used to generate the Poisson-surrogate were altered until a best fit could be made between the original and surrogate in terms of both power spectrum (B) and current/voltage distribution (C). Results displaying this goodness of fit for a single surrogate trace (red), as compared to the original (blue) are shown for both the 8-min voltage-clamp recording (Bi and Ci), and *in vivo* cat recording (Bii and Cii). (D) 50 Poisson surrogates were thus generated for both *in vitro* and *in vivo* recordings, and tested with the HRI detector, producing results for the *in vitro* mouse data (Di) and *in vivo* cat data (Dii). Traces in red represent the Poisson surrogate results, blue traces represent the HRI results from the original data, and the black dashed trace in Dii represents the 99% confidence interval for the Poisson surrogate results (analysis with the Jarque-Bera test of normality demonstrates that the rank ordered distributions of these scores are normally distributed for each rank order).

Surrogates were also created by implanting artificial postsynaptic currents (PSCs) or postsynaptic potentials (PSPs) in “plain” mother traces, the timing of which was determined by a Poisson number generator (Fig. 4). As in Mokeichev et al. (2007), the amplitudes and frequencies of these PSC/Ps were altered iteratively so that the power spectrum and current/voltage distribution of the surrogates were matched to the original traces (Fig. 4B). With regards to both the *in vitro* and *in vivo* data from Ikegaya et al. (2007), we again found that the original traces had more motif-repeats with higher HRI values (Fig. 4C).

### Recording conditions

**Figure 5:**
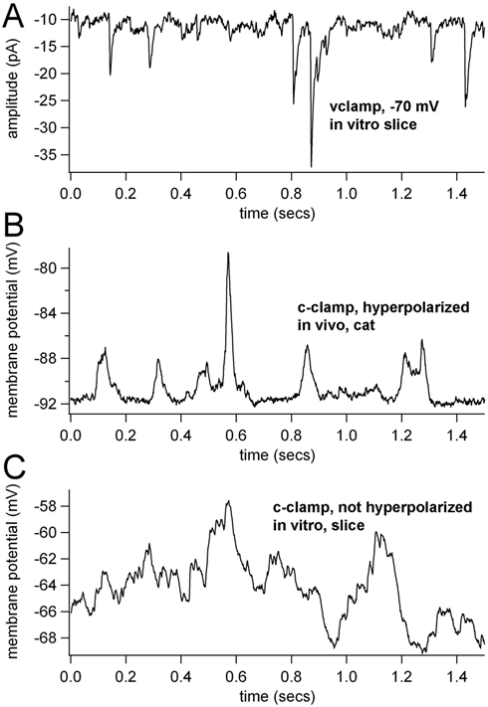
Intracellular recordings in different conditions. (A) Whole cell voltage clamp recording *in vitro* from a layer 5 pyramidal neuron, mouse V1 cortex. Vclamp = −70 mV. (B) Sharp electrode current clamp recording from cat visual cortex, *in vivo*, supragranular layer, with a large tonic hyperpolarizing current. (C) Current clamp recording from mouse cortex, *in vivo* and no tonic hyperpolarizing current. Note the similarities in recordings from *A* and *B* and how they both differ from *C*.

Inspection of the data themselves may yield some insights into why phase randomization results from the mouse *in vivo* recordings are so different from those of *in vitro* voltage clamp and cat *in vivo* recordings (Fig. 5). In both the cat *in vivo* recording and voltage clamp *in vitro* recording, we see stereotypical waveforms superimposed on a baseline, whereas in mouse *in vivo* recordings we see something that approximates colored noise. In the *in vitro* voltage clamp recordings these waveforms are putative PSCs. As for the cat *in vivo* recordings, they might be the result of very large PSPs, or perhaps the result of nearly synchronous PSPs. In either case, the single events themselves are stereotypical and repeatable, and the deterministic structure of these events is lost after phase randomization (Fig. 3, phase randomization traces). Therefore, it is not surprising that these surrogates would demonstrate a loss in repeatability compared to unshuffled traces.

### Interval shuffling

However, the fact that stereotypical single waveforms can be observed is not the main issue of contention in these studies—it is whether or not these waveforms, presumably driven by synaptic inputs from the neuronal network, can repeat in sequences of greater-than-chance precision. To address this issue, Ikegaya et al. (2004) identified the putative PSCs/PSPs using a correlation procedure (see methods) and pulled them out of the original recording, imposing them on a zero baseline. This procedure preserves the shape of the individual PSCs/PSPs as well as the timing of those events, creating an “extracted trace”. Surrogate traces were constructed from the extracted trace by shuffling the time intervals between the PSCs/PSPs, while preserving the temporal order of those events.

Mokeichev et al. (2007) argued that such a procedure could not be accomplished using the rat *in vivo* recordings because individual PSPs could not be reliably identified in most cases; we agree, and also confirm this finding with our mouse *in vivo* recordings. Mokeichev et al. (2007) further argued that our shuffling method may be too lenient in that trivial repeats comprised of just two PSCs/PSPs, possibly produced by the stereotypical firing pattern of a single presynaptic neuron, would be destroyed by our shuffling method; we agree with this argument as well. Their solution was to devise a shuffling technique that divided the intracellular recording into segments of approximately 400 msec. Surrogates were constructed by shuffling these segments. Thus, most of the two-event sequences are preserved in this manner.

A potential problem is that this shuffling procedure essentially shuffles the trace less thoroughly, and so the difference between surrogates and the original may not be detectable, even if deterministic repeats do exist. That is, the sensitivity of the detector program (i.e., the search program that finds repeats), may not be equipped for the task. Mokeichev et al. (2007) is aware of this caveat and tests it by injecting a 1 second long artificial repeat (i.e., absolutely deterministic) into the original recording, and then performs the 400 msec shuffling tests on this repeat-injected trace. They show that the detector does indeed distinguish the original with artificial repeat very well from the shuffled surrogates, arguing that the detector is sufficiently sensitive.

However, there is a significant problem with this sensitivity test: the LRI detector window itself is matched perfectly to the length of the artificial repeat (1 second). The basis of the detector algorithm is cross-covariance, and this function performs poorly if the detector window (set at 1 second in this program) does not match the actual length of the repeat to be detected. The original rationale for this sub-optimal detector (i.e., the LRI detection) is that it is merely a first-pass and saves much computation time. The actual values produced in the final analysis from HRI do not suffer from this defect since the detector window is matched to the width of the individual PSC (20 msec) or PSP (100 msec). Unfortunately, there can be many false positives from this 1^st^ pass in the detector algorithm such that many candidates never pass the threshold for gaining HRI analysis.

**Figure 6:**
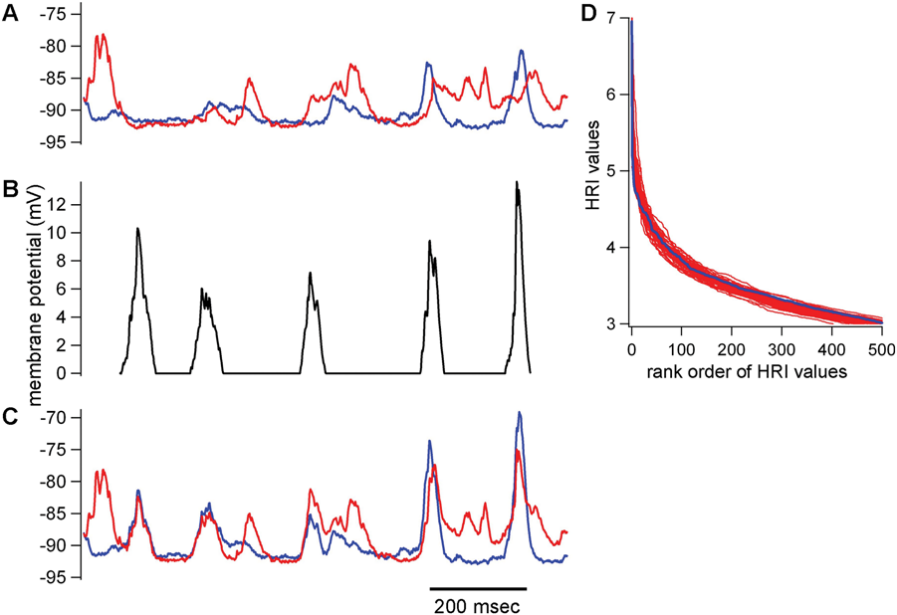
Implanting an artificial repeating motif into a shuffled recording. (A) A 400 msec shuffled surrogate from an original cat *in vivo* current clamp recording is composed. A one second segment from this shuffled surrogate recording is displayed (blue) with another one second segment from 9 seconds later superimposed (red). (B) The implant: a series of PSPs is constructed from the original recording, imposed on a 0 mV baseline. (C) The implant is summed into the 1 second segments, producing an implanted trace with recurring repeats. The implants are added approximate every 10 seconds into a 190 second recording, yielding 171 repeats. (D) Fifty 400 msec shuffle surrogates are constructed from the implanted recording, and the HRI values produced from those surrogates are compared to the values produced from the unshuffled implant recording. As shown, the LRI-HRI detection algorithm does not distinguish the implanted recording from the shuffled surrogates.

We demonstrated this defect in the detector program by implanting a motif that was not matched to the LRI detector window: the implanted motif was 850 msec, in contrast to the 1 sec detector window (Fig. 6). The implanted motif consisted of a series of 5 PSPs, and this motif was summed into a 400 msec interval shuffled surrogate from a 190 second cat *in vivo* current clamp recording. This implanted motif was inserted every 10 seconds, yielding 171 motif-repeat pairs. This implanted trace was then shuffled using the 400 msec interval shuffling technique, producing 50 surrogate traces. Using the LRI-HRI detector program, no difference could be found between the implanted trace and its shuffled surrogates (Fig. 6).

### Creating a better detector

**Figure 7:**
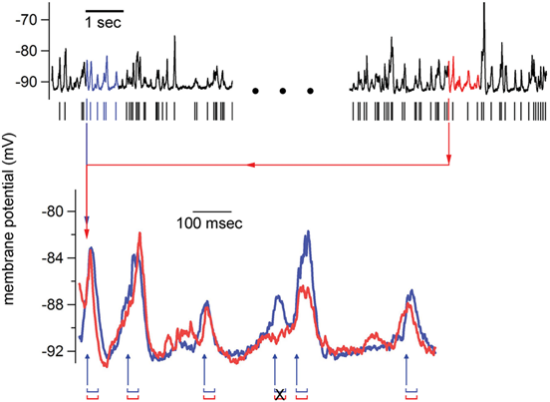
Repeat detection with PHRI. The onset times of putative PSPs are estimated by calculating all cross-covariance values of an average risetime waveform against the entire recording (190 seconds, *in vivo* cat, current clamp recording, hyperpolarized). This yields correlation values for every point in the recording, and those points with a high cross-covariance value and minimum amplitude are marked as onset time of a PSP, as shown above by the tally marks below the recording. These onset times comprise the comparisons that will be performed: *n* onset times yields n(n−1)/2 comparisons. One such comparison is shown above: two putative PSPs are identified with the longest blue tally and longest red tally. These PSPs are then aligned such that they yield the highest T value (using 30 msec rather than 100 msec window, see eq. 2). This alignment is preserved with respect to the comparisons made between the subsequent PSPs in each respective trace extracted from the recording. The T values are calculated for the intervals dictated by the PSP onset times in the motif trace (blue), indicated with blue arrows. T values below a set threshold are discarded from the HRI calculation, thus the black “X” indicating its non-incorporation into the HRI calculation. The minimum and maximum lengths of the motif-repeat traces that are included in the HRI calculation are 800 and 1200 msec, respectively. The minimum number of T values required for an HRI calculation are 3 (same as LRI-HRI criteria), and HRI is calculated as per eq. 3. The HRI values for all lengths between 800 and 1200 msec are calculated, and the motif-repeat length that yields the highest HRI value is saved. In the above example, the length of the motif and repeat is 853 msec, the PHRI = 5.3, and the delay between the motif and repeat is approximately 42 seconds.

In response to these results, we strived to create a detector program that could detect implanted repeats in the face of the 400 msec interval shuffle test. The goal was to create a detector that does not identify putative repeats with an arbitrary 1 second LRI window. Instead, putative repeats were detected by the onset times of PSPs. This new detector, PHRI (PSP-based detection, High Resolution Index), identifies the onsets of PSPs by their stereotypical risetimes, and then uses those PSP onset times as the pointers for the subsequent HRI analysis (Fig. 7). That is, every identified PSP is used as a point of alignment for a motif-repeat pair; the two selected PSPs, occurring at disparate times in the recording are aligned, and the trace that follows each is included as the motif-repeat pair to be examined. The 190 second long *in vivo* cat recording used in Fig. 7 contained 1351 identified PSPs, yielding 911925 motif-repeat pairs to be examined for subsequent HRI analysis—more than 100× the number of pairs identified with LRI analysis (6750 pairs). In order to reduce this substantial increase in computation time, the HRI analysis in PHRI is reduced by computing T values only for the regions identified as having PSPs (Fig. 7). In contrast, the LRI-HRI technique measures T values for every 1 msec interval of the 1 second trace (yielding 900 T value calculations).

As in the previous LRI-HRI technique, the criterion for a motif-repeat pair to pass HRI analysis is that it contains at least 3 regions where the T values exceed a minimum threshold. For the PHRI technique, the length of the motif-repeat is constrained to being at least 800 msec and no more than 1200 msec. The final length of the motif-repeat is defined as the length that yields the highest HRI value, and the mean length of the 10 best repeats from the cat *in vivo* trace in Fig. 7, using PHRI, is 933±32 msec.

**Figure 8:**
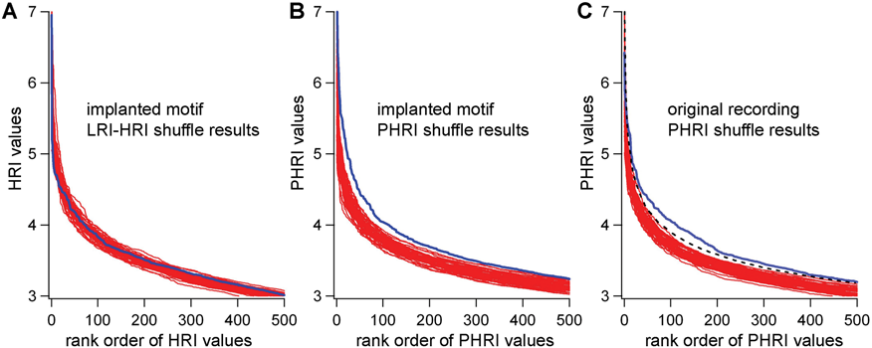
The improved detector finds implanted motifs and distinguishes the original recording from its 400 msec shuffled surrogates. (A) The original LRI-HRI detector is unable to distinguish the implanted recording from its shuffled surrogates. (B) The PHRI detector, applied to the same data set as *A*, appears to distinguish the unshuffled (blue) from the shuffled surrogates (red). (C) The original 190 sec cat *in vivo* current clamp recording and fifty 400 msec shuffle surrogates are examined with the PHRI detector. The rank ordered values from the original are shown in blue, and shuffled surrogates in red. As these values were normally distributed for each rank order, it was possible to construct confidence intervals for the distribution, and the 99% confidence interval is shown (dashed black line). The original recording results (blue line) are distinguished from the 99% confidence interval (p<0.01).

The various parameters of the PHRI analysis were varied in order to enable it to distinguish the implanted trace (see Fig. 6) from shuffled surrogates. When comparing PHRI values from 50 shuffled surrogates of the implanted trace to those of the unshuffled implanted trace there appears to be a significant difference in the distribution (Fig. 8B), or at least a much great difference in the difference compared to results obtained with the LRI-HRI method (Fig. 8A).

**Figure 9:**
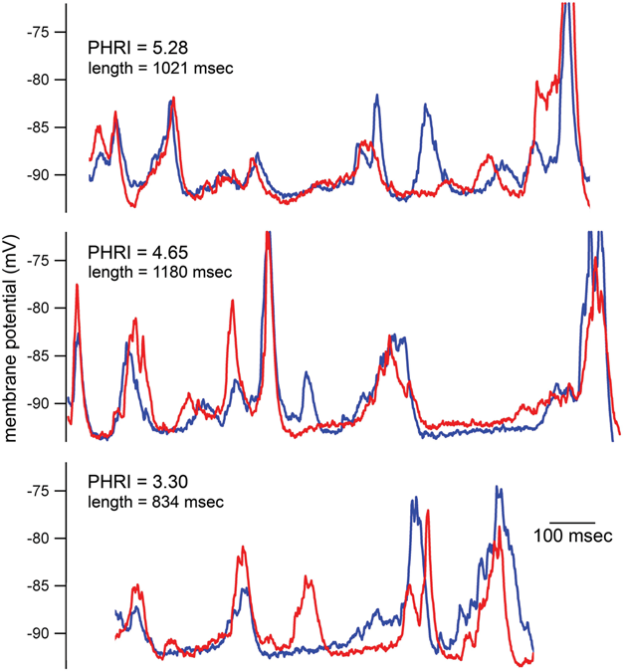
Three examples of repeats found using the PHRI detector from a 190 second long cat *in vivo* current clamp recording. Each motif-repeat example is labeled with its respective PHRI and its length. The PHRI values are a subset of those that make up the full set of PHRI values for this recording that are displayed in Fig. 8c.

We then used the PHRI analysis with the original 190 second recording, computing PHRI values from the original and fifty 400 msec interval shuffled surrogates. The rank ordered distributions of these scores are normally distributed for each rank order (using Jarque-Bera test of normality), allowing confidence intervals to be computed. As shown, the distribution of the PHRI scores for the original recording is outside the 99% confidence interval computed from the 50 shuffled surrogates (Fig. 8C). Motif-repeat examples from the original recording, selected from a range of PHRI values, are displayed in Figure 9.

### Revisiting recording conditions: an experiment

**Figure 10:**
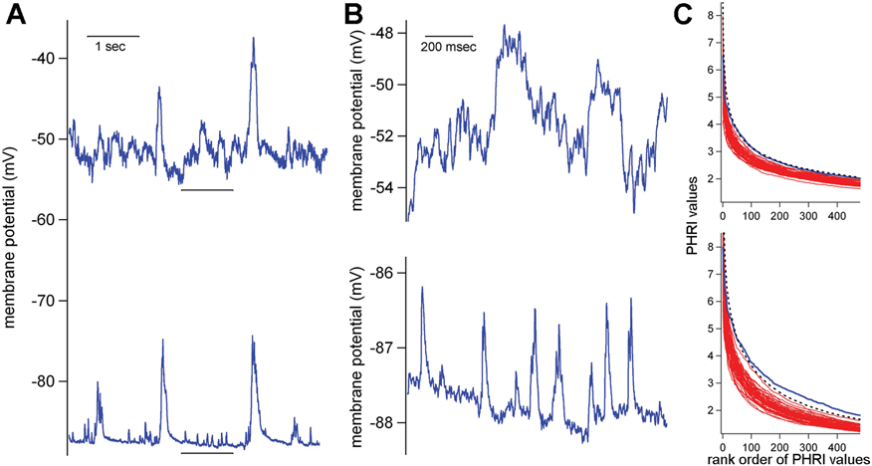
Effect of membrane potential hyperpolarization on PSP detection. (A) A sample of an *in vivo* current clamp recording is shown at an approximate resting membrane potential of −55 mV (top) versus a sample recorded from the same neuron minutes later at −95 mV (bottom). The hyperpolarized membrane potential was induced by a tonic DC injection. Underlined segments of these recordings are expanded in (B), showing the increased ability to detect smaller PSPs during hyperpolarized membrane potentials. (C) PHRI values recorded during resting membrane potential versus artificially induced hyperpolarized membrane potentials. (top) PHRI values are calculated from 90 seconds of resting membrane potential recording (mean −55 mV) (blue trace) and compared to 50 shuffle surrogates (red traces). Black dashed line represents the 99.9% confidence interval for the 50 shuffle surrogates. (bottom) The same results are shown, but with regards to 90 seconds of recording at hyperpolarized membrane potential (mean −90 mV), from the same neuron. The PHRI values obtained from the hyperpolarized section of recording (bottom) were significantly greater than the PHRI values obtained from the resting membrane potential recording (top) (p<0.001, Kolmogorov-Smirnoff test).

Having convinced ourselves that some *in vivo* and *in vitro* recordings show evidence of significantly repeating patterns, we then addressed a previously discussed hypothesis: repeats of synaptic inputs are better detected during hyperpolarized membrane potentials. To this end, we recorded a neuron from mouse somatosensory cortex, *in vivo*, in current clamp at approximately −60 mV resting membrane potential, and then applied a DC hyperpolarizing current, bringing the membrane potential to approximately −90 mV. This particular recording allowed the identification of some PSPs at −60 mV, but PSPs appeared to be more easily detectable at −90 mV (Fig. 10A,B). Both HRI and PHRI analysis yielded significantly higher values for the −90 mV section of the recording versus the −60 mV section (p<0.01 Kolmogorov-Smirnoff tests for each analysis, 100 seconds of recording in −60 mV and −90 mV). Furthermore, when fifty 400 msec shuffled surrogates were created for each, the −90 mV section of the recording exceeded the distribution by a significantly greater margin, exceeding 99.9% confidence intervals (Fig. 10C).

## Discussion

Based on our new analysis and data, we believe that the basic conclusions from Ikegaya et al. (2004) with regards to the analysis of the intracellular recordings remain valid. The data presented in that paper pass the surrogate tests presented in Mokeichev et al. (2007), although with the caveat that the repeat detector used in both of those papers was problematic. We further conclude that the phase randomization surrogates do not advance this study for generating surrogates, given the fact that they destroy the PSC/PSP structure of intracellular recordings.

We also conclude that the Poisson surrogates do not offer insight into the root question of this study. Unlike the phase randomization surrogates, they can at least imitate PSC/PSPs and thus retain these inherent, short repeats. In generating these surrogates, we reached the same conclusion as in Ikegaya et al. (2004), i.e., the surrogates contain significantly fewer repeats than the original. However, the argument against the surrogate data in Ikegaya et al. (2004) can also be used against these surrogates: the possibly trivial two consecutive PSC/PSP sequences are not conserved. Therefore, we conclude that the Poisson surrogates in this study do not resolve the root controversy.

It may be worth noting that it is much easier to generate Poisson surrogates that are very well-matched to original traces from *in vitro*, voltage-clamp recordings, as compared to *in vivo* current clamp recordings, as seen in the power spectrum and voltage distribution (Figs. 4B and 4C). This may not be surprising as the current clamp recordings should contain more intrinsic voltage-gated responses that require a more complicated model than that offered in this study. Indeed, the match in power spectrum and voltage distributions from the Poisson surrogates generated in Mokeichev et al. (2007) appear to be ill-matched to the original recordings (Fig. S2, Mokeichev et al., 2007). Furthermore, power spectrum and amplitude distributions are only two means of matching surrogates to original, and may exclude other important qualities of the original data. These problems in matching Poisson surrogate data to original data further undermine the results of this technique.

However, the 400 msec interval shuffling technique offered by Mokeichev et al. (2007) is a significant advance, and we focus on this particular surrogate generating technique for the remainder of the discussion. The interval shuffling technique inspired a re-examination of the original study and detection technique, and we discovered some flaws in the latter. In particular, the stated average repeat length of approximately 1 second reported in Ikegaya et al. (2004) should not be considered the true average length of repeats found intracellularly, but rather, an artifice resulting from the repeat detector program itself. That is, the initial LRI search in the detector program looks for repeats that are 1 second in duration—the repeat length itself is predetermined by the initial search window, which, as stated in Ikegaya et al. (2004), was chosen arbitrarily. The actual lengths of deterministic repeats in these networks are not indicated by these methods. This is a shortcoming of the detector program we originally used.

Another shortcoming, as revealed to us by results from Mokeichev et al. (2007), is the fallibility of using cross-covariance as a measure for similarity. This flaw is demonstrated by the detector's inability to distinguish a trace with implanted repeats from shuffled surrogates (Fig. 6). This finding suggests that if the initial detector window (1 second) is not matched to length of the repeat (in this case, 850 msec), then a false-negative result can be produced. This shortcoming is not so much a problem in the HRI detection part of the algorithm as the detector window is matched to the length of the synaptic events (between 20 msec and 100 msec). However, as the HRI algorithm scans only those sections of the recording indicated by the LRI search, then the entire LRI-HRI detector is compromised by the failing in the LRI.

In order to address these concerns, we devised a new detector, PHRI (Fig. 7). This detector differed from the previous in two ways: (1) putative repeats, to be carried over to HRI analysis, are selected based on the onset times of PSPs; (2) the algorithm that scans the putative repeats, creating T values, does not measure every 100 msec interval, but rather measures each interval as indicated by the onset times of the PSPs (Fig. 7). As for (1), this prevents the mistakes invoked by using an a priori 1 second detector window, and (2) reduces greatly the number of T calculations, yielding a faster analysis. This new detector distinguishes the implanted trace from shuffled surrogates (Fig. 8). In addition, the original cat *in vivo* recording is distinguished from shuffled surrogates in the rankings of repeat indices found in those recordings (p<0.01).

However, the question remained, why are some intracellular recordings, such as *in vitro* voltage clamp recordings, or the cat *in vivo* current clamp recording presented here, so different from the other *in vivo* current clamp recordings? The original idea of our method was to record the activity of many neurons in a synaptic network by recording the intracellular activity of just one neuron embedded within that network. This idea is not tested if the synaptic events are not resolved. The blurring of synaptic events could occur during current clamp recordings as the intrinsic voltage responses of the neuron are allowed to influence the recording. In addition, sharp electrode recordings may not reveal smaller synaptic events, in comparison to whole-cell recordings, perhaps allowing even greater reduction of synaptic events relative to the intrinsic voltage fluctuations. It is also conceivable that this technique is inappropriate for *in vivo* recordings where the number of synaptic inputs is so great that resolving them individually is not feasible with a recording at the soma alone.

These speculations do not address the current clamp sharp electrode recording from cat cortex, *in vivo*, where significant repeats could be found using all shuffle surrogates tested. One feature of this recording that distinguishes it from the rat *in vivo* recordings so far reported is the large tonic hyperpolarizing current that was applied to the neuron. This current was applied to prevent action potentials from occurring, in accordance with the protocols from Lampl et al. (1999) [26]. It is conceivable that such a large hyperpolarizing current may prevent many voltage-gated channels from operating, especially as many of those channels are activated at more depolarized levels. In addition, it's possible that the neuron is held either at or hyperpolarized to the GABA-A reversal potential. Thus, all synaptic events are either strongly depolarizing or negligible, allowing a flat baseline upon which these currents may be resolved. We tested these ideas by recording a neuron from mouse somatosensory cortex *in vivo* in current clamp where half of the recording was at a membrane potential of about −60 mV, and the latter half at −90 mV. We demonstrate that the repeats found at −90 mV have a significantly greater distribution of repeat indices than those at −60 mV (p<0.001), and we show that this recording also has a greater distribution than its shuffled surrogates (p<0.001) (Fig. 10).

It has been argued that there is no method for surrogate testing of repeated patterns of spontaneous activity that will satisfy every researcher. Therefore, we believe that future studies of these phenomena should be more experimental in nature, addressing their mechanism and biological function. If these repeats are deterministic, then it should be possible to disrupt, manipulate or evoke them. Indeed, MacLean et al. (2005) [6], where intracellular repeats were actually evoked by thalamic stimulation, is an example of such an experiment, where the reality of the repeats was demonstrated, since they were generated by the stimulation. Without experiments that demonstrate deterministic origins, the biological significance of these repeat phenomena will remain a topic of contention.

## Materials and Methods

### Intracellular recordings

*In vitro* voltage clamp recordings and the cat intracellular *in vivo* recording are the same recordings that were analyzed in Ikegaya et al. (2004).

Mouse *in vitro* recordings: 8 minute long whole cell voltage clamp recordings were performed with 6–9 MΩ intracellular electrodes in large layer 5 pyramidal cells in mouse V1. Neurons were voltage clamped at −70 mV in standard ACSF (1 mM MgSO_4_, 2 mM CaCl_2_, 3 mM KCl, 34°C), and no stimulation was applied (spontaneous activity recorded). The coronal slices, 350 µm thick, were taken from P18–22 C57Bl/6 mice. Further details can be found in Ikegaya et al. (2007) [7].

Cat *in vivo* recordings: neurons in supragranular cortex in area V1 were recorded intracellularly with sharp electrodes filled with 2 M potassium acetate. The adult cats were paralyzed and barbiturate-anesthetized, and no stimulation was given during the recordings (spontaneous activity only). For the recording analyzed in this study, a tonic hyperpolarizing current was applied to prevent spontaneous action potentials, and the recording was stable for 10 minutes. Further details of the *in vivo* recordings can be found in Lampl et al. (1999) [26] and Chung and Ferster (1998) [27].

Mouse *in vivo* recordings: Postnatal day 17 to 23 ICR mice were anesthetized intraperitoneally (ip) with 1 g/kg Urethane or 50 mg/kg pentobarbital. The head was immobilized using a metal pedestal fastened to the skull. After a craniotomy either with a needle or forceps, the skull was covered with 2% agarose in 0.1 M phosphate buffer. Glass micropipettes (4–6 M) were filled with (in mM) 135 K-gluconate, 4 KCl, 0.1 Ca_2_Cl_2_, 0.4 Na_2_GTP, 4 MgATP, 1 EGTA and 10 HEPES (pH 7.2). The somatosensory or motor cortex was approached dorsocaudally at a 90° angle with the horizontal under high positive pressure, which was lowered to approximately 30 mBar at about 200 µm below the skull surface. Data were acquired with a MultiClamp 700A patch-clamp amplifier and pCLAMP 9 software (Axon Instruments). Potentials were filtered at 10 kHz (eight-pole Bessel filter) and sampled at 20 kHz with a 16-bit A/D converter (Digidata 1322A). These mouse *in vivo* experiments were performed under the approval of the animal experiment ethical committee at the University of Tokyo (approval number 19–35 and 19–41), according to the National Institute of Health guide for the care and use of laboratory animals.

### Analysis

#### Finding repeats of intracellular activity: LRI-HRI method

The technique discussed here uses intracellular recordings from single neurons as a means to “listen” to potentially all of the activity of all neurons that form synapses with that recorded neuron. As a single pyramidal neuron may receive 1000 s of synapses from other neurons, most of them locally, then this technique has the potential to yield information about a large fraction of a cortical column (Fig. 1). This procedure for finding repeats of intracellular activity has been described in Ikegaya et al. (2004) [7] as well as Mokeichev et al. (2007) [24]. There are two stages in the search for repeats: (1) a low resolution search, producing a low resolution index (LRI) and (2) a high resolution search, producing a high resolution index (HRI). Both methods are forms of template matching: two segments are isolated from a long recording and the similarity between those segments is quantified.

#### Low Resolution Index (LRI)

The LRI compares 1 second segments of the recorded waveform, using a nested loop of template matching (Fig. 2). The cross-covariance function is at the heart of this analysis, and this function quantifies the temporal similarities of the recorded waveforms.
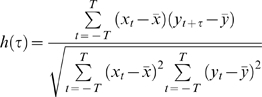
(1)


Here, x and y are amplitudes from the respective motif and its potential repeat, and 2T+1 are the number of samples in each motif at 1 point per msec. The length of x and y are 1 second (1000 points at 1 point per millisecond), and τ represents the lag time between x and y. The motifs and repeats are defined by these lengths and the incremental jump from one potential repeat to another is 250 msec (in Fig. 2 this would represent the incremental movements of the colored brackets). As jumps of 250 ms are unlikely to find the regions of precise overlap, the program realigns the traces according to the difference between the peak value of the covariance function and the zeroeth lag of this function (i.e., the value at τ = 0) and then recomputes the function, provided that the peak value is initially within 250 ms of the zeroeth lag. The value at the zeroeth lag (h(0)) is then recorded. The highest values for each 1 second interval and those passing a set threshold were collected for each recording and formed our low resolution similarity index (LRI). The threshold was set according to a level that yielded a reasonable number of putative motif-repeats that could be analyzed with subsequent HRI analysis. “Reasonable” is defined here as taking less than a few days of computation time with HRI analysis, and per recording this would mean on the order of 10000 putative repeats. In most recordings the threshold was set to approximately 0.45. In this sense, the thresholds here not considered definitive.

The 1 second length of the motif and repeats is also arbitrary, and, as discussed later, problematic. This initial identification is, however, somewhat justified in reducing what would otherwise be an overly burdensome computational task. That is, the LRI is used to identify putative repeats, remember the locations of those putative repeats, and then analyze more carefully those segments in subsequent analyses. Segments that do not pass a minimum threshold are passed over and not analyzed further, saving some time in the subsequent intensive analysis.

#### Hig Resolution Index (HRI)



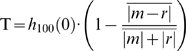
(2)

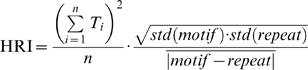
(3)


Those threshold-passing motif-repeats identified with the LRI are saved later for calculation of HRI. For HRI, the two 1 second segments are compared in greater detail as cross-covariance functions are computed for every 20 msec interval between the two 1 second segments (Fig. 2). This 20 msec interval is determined by the average width of a PSC. When recording PSPs in current clamp, the charging of the membrane results in longer synaptic signals, and in those cases 100 msec intervals are used. In both cases, it is important that the width of the cross-covariance window is matched to the mean estimated duration of an individual synaptic event. The HRI is computed from the number of threshold-passing 20 msec intervals, the similarity measured in each of those 20 msec intervals (T values, Eq. 2), as well as a general similarity index for the entire duration of the putative repeat (Eq. 3).

#### Finding repeats of intracellular activity: PHRI method

The newer method, PHRI, differs from the LRI-HRI method mostly in terms of how putative repeats are detected: rather than using cross-covariance of 1 second samples from the recording, the PHRI identifies potential repeats by the onset times of identified PSPs (or PSCs) (Fig. 7). The PSPs are identified by their risetimes in a method nearly identical to that from Ikegaya et al. (2004) with regards to the extraction of PSPs in that paper: PSPs were detected by computing a covariance function of a mean PSP rise time waveform (4–6 msec in duration) against the entire spontaneous recording: this produced a waveform whose peaks marked the onset of PSPs, and peaks passing a set threshold (typically, 0.9) were taken as the start times of PSPs. In some cases, an amplitude threshold was used in conjunction with the covariance function threshold. Thresholds were adjusted so that the fewest false positives and false negative results appeared, as can be judged in viewing Fig. 7. Importantly, the number of identified PSPs found in surrogate traces versus original traces was unchanged by the creation of 400 msec shuffled surrogate traces.

The identified onset times of PSPs were then used as the points of alignment for comparing two different stretches of a recording, called here a putative motif-repeat (Fig. 7). T values (Eq. 2) are then calculated at these aligned motif-repeats, but in contrast to LRI-HRI analysis, the T values are only calculated at the onset times of PSPs found in the motif of the motif-repeat. These T values are then used just as before in the calculation of HRI (Eq. 3). With this PHRI technique, the length of the motif-repeat is determined by length that yields the highest HRI value, and it is constrained by having a minimum of 800 msec and a maximum of 1200 msec. This constraint is enacted with respect to the shuffle surrogate technique described below: if motif-repeats are allowed that are the same length of the shuffle lengths (400 msec), then the shuffling is likely to keep many of the motif-repeats intact (it would be analogous to using the LRI-HRI technique and shuffling with 1000 msec segments). As in the LRI-HRI technique, a minimum of three T values that pass threshold are required.

#### Surrogate traces

The production of surrogate traces was performed using three methods described in Mokeichev et al. (2007), namely, phase randomization, Poisson simulation, and interval shuffling. The phase randomization technique performs a fast Fourier transform (FFT) of the original trace, decomposing it into its frequency components. There is a particular phase and amplitude associate with each component, and in this shuffle technique the original phases are replaced with randomly chosen phases. After a reverse FFT, a surrogate trace is produced where the temporal relationships between its various frequency components have been randomized with respect to the original, while the frequency power spectrum remains the same.

The Poisson simulation creates surrogate traces by stimulating a single model neuron with synaptic inputs. The relative strengths and frequencies of these inputs are manipulated so that the simulated recording in the model neuron produces a surrogate trace that is similar with respect to the original with regards to power spectrum and voltage (or current) distribution.

The interval shuffling protocol arguably randomizes the original trace the least thoroughly, and so is the most rigorous shuffling protocol. In this, the original trace is “cut” into segments of approximately 400 msec long. These segments are then randomly reattached to each other, with certain constraints so that no artificially abrupt changes in voltage are introduced. The “cut points” are determined by two different voltage levels that are chosen according to the lower third and upper third of the total voltage amplitude distribution. This is a shuffling in the time domain, and produces surrogates with the same power spectrum and voltage distribution[24].

The surrogate trace generation techniques were implemented using Igor software (Wavemetrics). The search for repeats in these and original traces were conducted using Matlab (Mathworks) software on a 288-unit cluster computer.
